# Anti-Swelling Antibacterial Hydrogels Based on Electrostatic Repulsion and Hydrophobic Interactions for Human Motion Sensing

**DOI:** 10.3390/jfb16090346

**Published:** 2025-09-14

**Authors:** Zexing Deng, Litong Shen, Qiwei Cheng, Ying Li, Tianming Du, Xin Zhao

**Affiliations:** 1 College of Materials Science and Engineering, Xi’an University of Science and Technology, Xi’an 710054, China; biomaterial@xust.edu.cn (Z.D.); 24211225047@stu.xust.edu.cn (L.S.);; 2 Beijing International Science and Technology Cooperation Base for Intelligent Physiological Measurement and Clinical Transformation, Department of Biomedical Engineering, College of Chemistry and Life Science, Beijing University of Technology, Beijing 100124, China; 3 State Key Laboratory for Mechanical Behavior of Materials, Xi’an Jiaotong University, Xi’an 710049, China

**Keywords:** zwitterionic polymers, anti-swelling hydrogels, antibacterial hydrogels, strain sensors

## Abstract

The development of high-performance sensing materials is critical for advancing bioelectronics. Conductive hydrogels, with their unique flexibility, are promising candidates for biomedical sensors. However, traditional conductive hydrogels often suffer from excessive swelling and undesirable antibacterial activity, limiting their practical use. To overcome these challenges, anti-swelling, antibacterial, and ionically conductive hydrogels were built through free radical polymerization. The preparation was conducted using a monomer mixture comprising acrylic acid (AA), the antibacterial zwitterionic compound [2-(methacryloyloxy)ethyl]dimethyl-(3-sulfopropyl)ammonium hydroxide (SBMA), and the hydrophobic monomer lauryl methacrylate (LMA). The protonation of SBMA by AA enables electrostatic repulsion, thereby imparting anti-swelling properties to the hydrogel. The introduction of hydrophobic LMA components further enhances the anti-swelling and mechanical performance of hydrogel. The resulting hydrogel exhibits excellent anti-swelling property with a swelling ratio of 59.36% after 120 h and good mechanical performance with a tensile strength of 158 kPa, an elongation at break of 176%, and a compressive strength of 0.37 MPa at 80% strain. In addition, hydrogels possess superior sensing performance for strain sensing with a gauge factor of 1.315 within 40–60% of strain, 330 ms of response time, and 177 ms of recovery time. Furthermore, the hydrogel is capable of monitoring human motion and physiological signals. These attributes make it highly suitable for wearable sensors and biomedical monitoring applications.

## 1. Introduction

Gels are a kind of dilute cross-linked material which have been extensively utilized in the biomedical field, food industry, daily chemistry industry, etc. [1,2,3,4]. Ionic conductive hydrogel-based sensors have found widespread applications in motion monitoring [5,6], healthcare diagnostics [7,8], and electronic skin systems [9,10,11], especially integrated with artificial intelligence (AI) signal process systems in recent years [12,13,14], owing to their outstanding combination of mechanical softness [15,16], high flexibility [17], potential biocompatibility [18,19], and optical transparency [20]. Nevertheless, a critical challenge persists regarding their structural stability in aqueous environments, where excessive water absorption and subsequent swelling behavior frequently lead to network and function disruption, ultimately compromising the reliability and performance of ionic hydrogels in practical scenarios [21,22,23]. This fundamental limitation highlights the need for developing novel ion-conducting hydrogels with enhanced anti-swelling properties.

To address the swelling issue of hydrogels, researchers have proposed various strategies, including the construction of multiple crosslinking networks [24,25], the introduction of electrostatic repulsion forces [26,27], and additional hydrophobic components [28]. For instance, poly(2-hydroxyethyl methacrylate) (PHEMA) hydrogels are hydrophilic. The cationic monomer diallyldimethylammonium chloride (DDA) can be incorporated to construct an anti-swelling hydrogel P(HEMA-co-DDA) by leveraging electrostatic repulsion [29]. Additionally, 2-acrylamido-2-methylpropanesulfonic acid (AMPS), hydroxyethyl methacrylate (HEMA), and N, N’-methylenebisacrylamide (BIS) were polymerized in ethylene glycol (EG). Subsequent solvent exchange facilitated hydrogen bonding between sulfonic acid and hydroxyl groups, thereby conferring the P(AMPS/HEMA/EG) hydrogel with outstanding mechanical performance. By adjusting the ratio of AMPS to HEMA, a hydrophilic–hydrophobic segment balance was achieved, thereby reducing water molecule infiltration into the network structure and minimizing swelling [30]. These findings demonstrate the considerable promise of anti-swelling conductive hydrogels in expanding their application scope. However, research on anti-swelling hydrogels constructed based on multiple crosslinking strategies remains limited. Combining multiple approaches is expected to yield hydrogels with superior anti-swelling properties.

[2-(Methacryloyloxy)ethyl]dimethyl-(3-sulfopropyl)ammonium hydroxide (SBMA) is an attractive zwitterionic monomer with significant potential for various applications. Its molecular architecture contains both positively charged quaternary ammonium groups and negatively charged sulfonate groups, enabling unique advantages in hydrogel design [31,32]. The dissociated ions from SBMA can freely migrate through the hydrated polymer network, establishing efficient ionic conduction pathways that are independent of external electrolytes [33,34]. Moreover, SBMA-based hydrogels demonstrate exceptional resistance to salt precipitation while maintaining stable conductivity across varying electrolyte concentrations [35]. In addition, they can be built for smart electronic devices at the human tissue–device interface [36,37]. Another significant benefit is SBMA’s ability to minimize non-specific protein adsorption while enhancing biocompatibility—characteristics that are particularly valuable for biomedical applications [38,39]. These combined properties establish SBMA as an excellent candidate material for developing advanced flexible ionic conductive hydrogels. For instance, an anti-swelling ionic conductive hydrogel was successfully fabricated using poly(vinyl alcohol) (PVA) and a copolymer composed of SBMA and 2-hydroxyethyl methacrylate (HEMA) (P(SBMA-co-HEMA)) [40]. Furthermore, lauryl methacrylate (LMA), a representative long-chain alkyl acrylate, not only confers strong hydrophobicity to the molecular structure through its alkyl chains but also participates in free radical polymerization via its unsaturated double bonds to construct the network backbone [41,42,43]. Research has revealed that the hydrophobic interactions of LMA can effectively suppress water molecule penetration while enhancing the mechanical strength of hydrogels [44,45]. As an example, a multifunctional eutectic gel exhibiting high transparency, anti-freezing, anti-swelling, and self-healing capacities was developed through the photopolymerization of acrylic acid (AA) and LMA [46].

In this study, anti-swelling ionic conductive hydrogels were fabricated based on acrylic acid (AA), LMA, and SBMA. The poly(AA-co-SBMA) hydrogels showed anti-swelling and antibacterial performance because of electrostatic repulsion, hydrophobic interactions, and quaternary ammonium moieties. Furthermore, the incorporation of hydrophobic LMA monomers effectively optimized both the mechanical and anti-swelling properties of the final poly(AA-co-LMA-co-SBMA) hydrogels, yielding outstanding performance characteristics including a swelling ratio of 59.36% after 120 h immersion in deionized water, a tensile strength of 158 kPa, an elongation at break of 176%, and a compressive strength of 0.37 MPa at 80% strain. The hydrogel also exhibited remarkable strain-sensing capability with a gauge factor of 1.315 within the 40−60% strain range. Furthermore, the prepared poly(AA-co-LMA-co-SBMA) hydrogels can be used in human motion and physiological signal detection, demonstrating significant potential for advancing hydrogel sensors.

## 2. Experimental Section

### 2.1. Materials

[2-(methacryloyloxy)ethyl]dimethyl-(3-sulfopropyl)ammonium hydroxide (SBMA) and acrylic acid (AA) were obtained from Macklin (Shanghai, China). Lauryl methacrylate (LMA) was sourced from Aladdin (Shanghai, China). N, N’-methylenebisacrylamide (BIS) and ammonium persulfate (APS) were procured from Sigma-Aldrich (Shanghai, China). All chemicals employed were of analytical grade.

### 2.2. Preparation of Hydrogels

The hydrogels were developed through free radical polymerization. First, the different weights or volumes of monomers shown in Table 1 were weighed. For each group, 1 mL of deionized water was added and stirred for 0.5 h to obtain a homogeneous sediment-free solution. Then, APS (1 wt.%) and BIS (0.5 wt.%) solutions were added to each group and mixed well. Finally, the solutions were transferred into centrifuge tubes and placed in an oven for polymerization and crosslinking at 60 °C for 5 h. The specific contents of each component in the hydrogels are shown in the table below (Table 1).

### 2.3. Characterization of Hydrogels

The structure of the hydrogels was systematically characterized using multiple analytical techniques. Fourier-transform infrared spectroscopy (FT-IR; PerkinElmer Spectrum Two, Shelton, CT, USA) was conducted on hydrogel samples to elucidate their chemical composition and structure. Additionally, the morphology of both pristine and swollen hydrogels was visualized by a scanning electronic microscope (SEM; Hitachi S4800, Osaka, Japan). Before observation, the hydrogel specimens were sputter-coated with a gold layer.

### 2.4. Mechanical Properties of Hydrogels

The mechanical performance of the hydrogels was comprehensively evaluated through rheological and mechanical testing. The rheological properties of hydrogels (Ø20 mm × ~1 mm) were analyzed using a rheometer (TA Instruments, Discovery HR 20, New Castle, DE, USA) to determine the angular frequency dependence of the storage modulus (G’) and loss modulus (G”). A universal testing machine (LD23, 50 N load cell, Shanghai, China) was employed to conduct uniaxial tensile tests on rectangular hydrogel samples (25 × 4 × 3 mm^3^). The tests were conducted at a constant crosshead displacement rate of 10 mm/min. Additionally, cyclic loading tests were carried out at a speed of 50 mm/min. Compression testing employed cylindrical samples (Ø50 mm × 30 mm) deformed at 80 mm/min. All mechanical tests were conducted in triplicate to obtain reliable and reproducible results.

### 2.5. Anti-Swelling Properties of Hydrogels

The swelling stability of the hydrogels was quantitatively assessed using gravimetric swelling measurements. Hydrogel samples were first weighed to determine their initial mass (W_0_), followed by immersion in deionized water at room temperature. At predetermined time intervals, the surface moisture of samples was gently removed by a filter paper, and the swollen mass (W_s_) of samples was immediately measured. The swelling ratio (SR) was obtained from the equation below:

SR = (W_s_ − W_0_)/W_0_ × 100% (1)

### 2.6. Antibacterial Activities of Hydrogels

The antibacterial properties of the hydrogel were evaluated using representative *S. aureus* (Gram-positive) and *E. coli* (Gram-negative) strains. Experimental protocols involved adding 0.1 mL bacterial suspensions (OD_600_ = 1.0) to 24-well plates containing either 0.2 mL of phosphate-buffered saline (PBS blank control group) or hydrogel samples (experimental group). Following 1 h incubation at 37 °C, the bacterial solutions were diluted with 0.9 mL PBS, and 0.1 mL aliquots were plated on agar medium. After 24 h cultivation, antimicrobial efficacy was quantitatively determined by colony-forming unit (CFU) enumeration.

### 2.7. Biocompatibility Properties of Hydrogels

The cytocompatibility of the hydrogels was evaluated using a direct contact test with L929 mouse fibroblast cells from the Chinese Academy of Sciences. The cells were pre-cultured in 96-well plates for 24 h using DMEM supplemented with 10% newborn calf serum (NBCS) under standard conditions (37 °C, 5% CO_2_). Following PBS washing, test groups were exposed to hydrogel specimens, while controls received no treatment. Medium refreshment was performed every 48 h during the experiment. Detailed experimental procedures can be found in our previous study [47].

### 2.8. Sensing Properties of Hydrogels

This human study conformed to the principles of the Declaration of Helsinki and received prior approval from the Xi’an Jiaotong University Ethics Committee (XJTUAF2024LSYY-081). To monitor human body movements, the hydrogel sensor was attached to the skin surface at target locations. Adhesive tapes were used to fix the hydrogels during the sensing process to ensure stable acquisition of physiological signals. Copper wire-connected probe electrodes were attached to both ends of the hydrogel and linked to a digital multimeter for continuous signal monitoring. A uniform silicone oil coating was applied to the hydrogel surface to minimize water evaporation during measurements. The relative resistance change (ΔR/R_0_) was calculated in real-time to quantify motion-induced deformations, and the relative resistance variation was determined using the following definition:

∆R/R_0_ = (R − R_0_)/R_0_ × 100% (2)

where R_0_ represents the initial resistance and R denotes the real-time resistance during deformation.

The strain sensitivity, quantitatively characterized by the gauge factor (GF), is defined as the relative change in electrical resistance (ΔR/R_0_) per unit applied strain (ε), expressed mathematically as the following equation:

GF = (ΔR/R_0_)/ε (3)

where ε represents the externally applied mechanical strain.

## 3. Results and Discussion

### 3.1. Preparation of Hydrogels

**Scheme 1:**
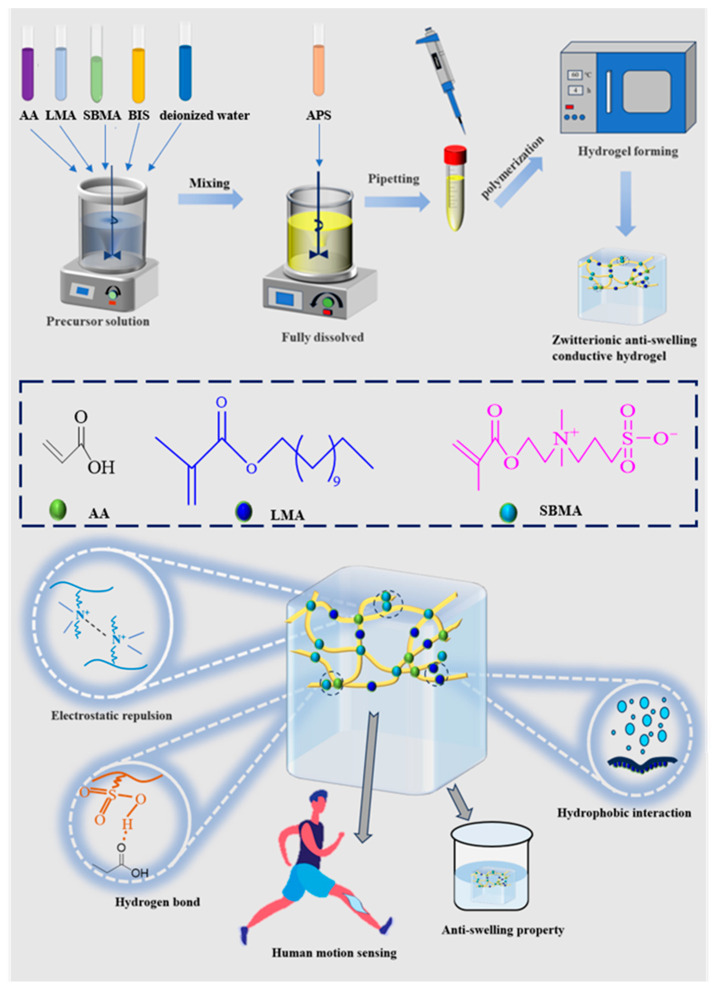
Fabrication and mechanistic overview of the A-L-S hydrogel.

As illustrated in Scheme 1, the hydrogel was initially prepared through the dissolution of AA, SBMA, and LMA monomers, followed by the addition of APS initiator and BIS crosslinker to the homogeneous solution. Thereafter, the mixture was transferred into centrifuge tubes for polymerization. This fabrication process enhanced intermolecular interactions through hydrogen bonding and electrostatic repulsion forces between AA and the zwitterionic SBMA, benefiting the anti-swelling property of hydrogels. Furthermore, the incorporation of a hydrophobic LMA monomer concurrently could optimize both the mechanical properties and hydrophobicity of the resulting hydrogel network. As a result, the anti-swelling antibacterial hydrogels were successfully built.

### 3.2. Characterization of Hydrogels

**Figure 1:**
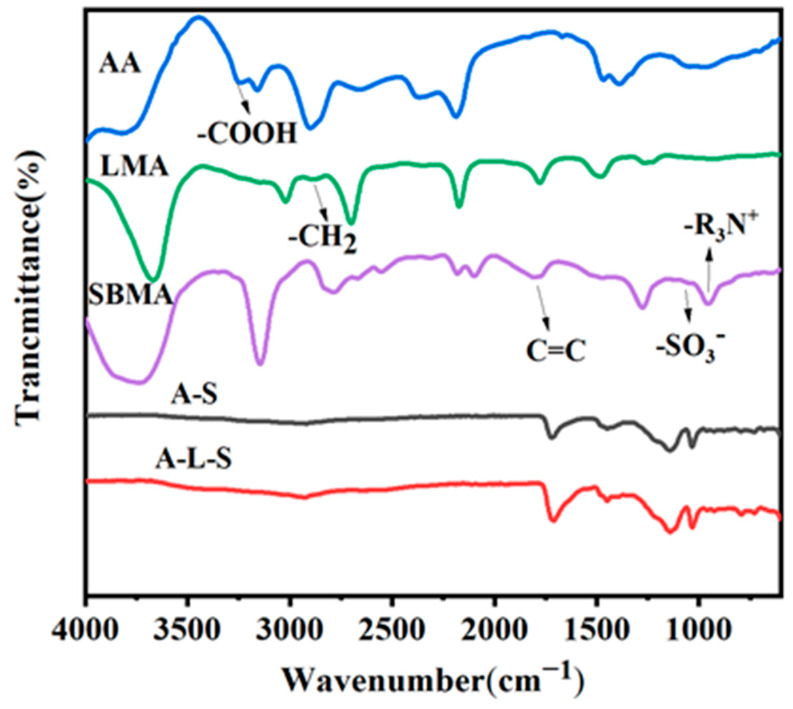
FT-IR spectral analysis of AA, LMA, SBMA, A-S, and A-L-S.

The molecular architecture of the poly(AA-co-LMA-co-SBMA) (A-L-S) hydrogel was characterized by FT-IR. As shown in Figure 1, the spectrum revealed characteristic absorption bands at 3250 cm^−1^, corresponding to the -COOH stretching vibration of AA. A peak at 2850 cm^−1^ (-CH_2_ stretching vibration) was ascribed to the characteristic absorption peaks of LMA, confirming its long alkane chain structure. Peaks at 1038 cm^−1^ (-SO_3_^−^ stretching vibration), 953 cm^−1^ (N^+^-C stretching vibration), and 1656 cm^−1^ (C=C stretching vibration) were assigned to the featuring peaks for SBMA. Notably, apart from the disappearance of C=C peaks, the newly found peaks at 1100–1300 cm^−1^ belonged to the characteristic peaks of C-C bonds. Meanwhile, other characteristic peaks of LMA and SBMA remained, indicating the preparation of an A-L-S hydrogel.

**Figure 2:**
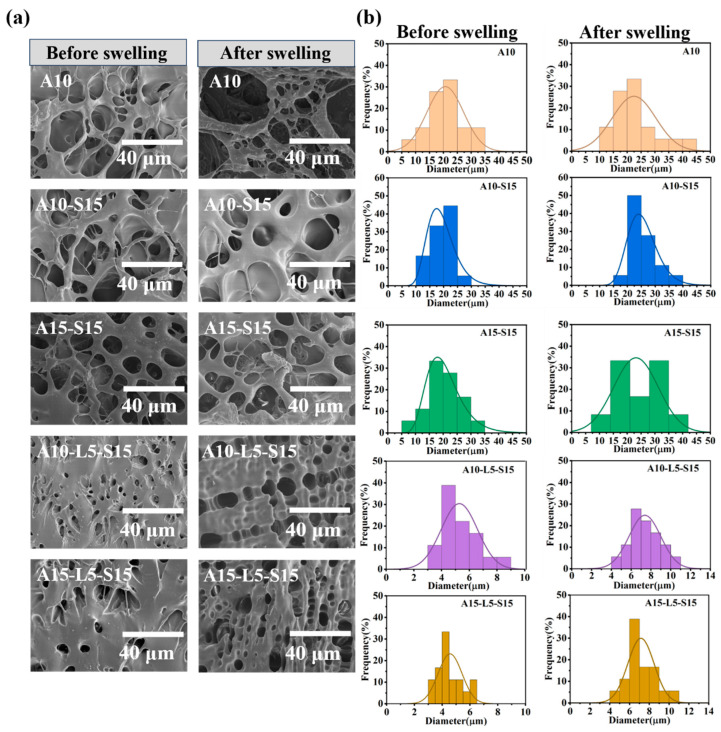
Swelling behavior and structural characterization of AA, A-S, and A-L-S hydrogels. (**a**) SEM images and (**b**) pore size distribution profiles before and after swelling.

The microstructural evolution of hydrogels was systematically investigated through scanning electron microscopy (SEM) analysis (Figure 2a). A microstructural analysis of cross-sections indicated that all formulated hydrogels preserved porous network structures. Before swelling, the pore size of hydrogels decreased with the increased concentration of monomers, especially after the introduction of hydrophobic LMA. This is because an increased crosslinking ratio would lead to a decreased pore size.

After swelling, the pore size change also demonstrated a similar pattern with an increased concentration of monomers. Post-swelling analysis demonstrated significant structural differences. While pure poly(acrylic acid) (AA) hydrogels exhibited substantial pore expansion after 120 h immersion in deionized water due to their inherent hydrophilicity, the SBMA and LMA modified variants displayed markedly smaller pore size. This microstructural refinement became particularly evident when increasing the AA content from 100 μL to 150 μL, where enhanced hydrogen bonding and electrostatic interactions combined with hydrophobic interaction contributions from LMA’s alkyl chain organization synergistically produced more compact hydrogel networks.

The structural stability of hydrogels under swelling conditions was evaluated by monitoring pore size distribution (Figure 2b). Statistical evaluation revealed distinct swelling behavior among AA, A-S, and A-L-S hydrogels. The AA hydrogel underwent substantial pore expansion after swelling, while the A-L hydrogels and A-L-S hydrogels maintained remarkable dimensional stability with negligible pore size variation post-swelling. This pronounced anti-swelling performance of A-L-S hydrogels was further corroborated by the SEM morphological observations, demonstrating exceptional structural integrity after water immersion.

### 3.3. Mechanical Properties of Hydrogels

**Figure 3:**
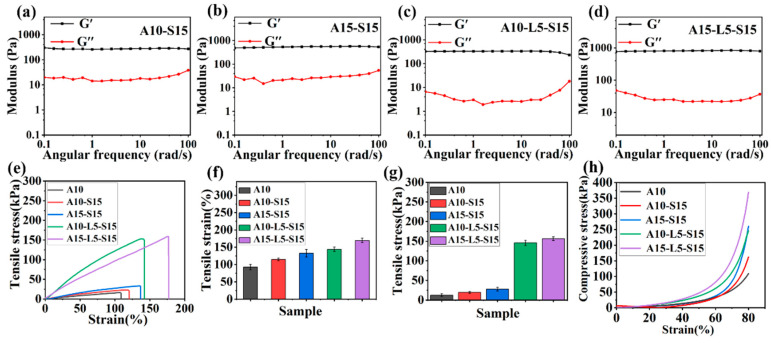
Rheological and mechanical characterization of hydrogels. Rheological property of (**a**) A10-S15, (**b**) A15-S15, (**c**) A10-L5-S15, and (**d**) A15-L5-S15. (**e**) Tensile stress–strain curve. (**f**) Elongation at break values. (**g**) Tensile strength values. (**h**) Compressive stress–strain curves.

Hydrogels are not ideal, not being purely elastic solids or purely viscous liquids. They are viscoelastic materials, exhibiting both the elasticity of a solid and the viscosity of a liquid. Rheological oscillation testing is the gold standard for characterizing this complex behavior. Generally speaking, the storage modulus (G’) measures the elastic or solid nature of a hydrogel, which is its ability to store energy and resist deformation. The loss modulus (G”) measures the viscous or liquid nature of a hydrogel, which is its ability to dissipate energy and flow. All A-S and A-L-S hydrogels demonstrate a G’ > G” state when the angular frequency is 0.1 to 100 rad/s (Figure 3a–d). This means that energy is primarily stored and the material can recover its original shape after being subjected to external forces. This is a typical characteristic of a formed hydrogel. When G’ < G”, the material has stronger flow characteristics. The internal structure is weak or unstable, making it more susceptible to irreversible flow and destruction under external forces. This is usually the state of a sol solution. A high G’ value indicates a high crosslink density, where the molecular chains are tightly connected and thus very resistant to deformation. A low G’ value indicates that the cross-linking density is low or the molecular chain itself is relatively soft and easily deformed. The G’ of A15-S15 and A15-L5-S15 was higher than that of A10-S15 and A10-L5-S15, respectively. Mechanical tensile behavior evaluation revealed substantial composition-dependent mechanical performance. The pure AA hydrogel exhibited limited mechanical performances, demonstrating a tensile strength of 10 kPa and elongation at break of 94%. The addition of zwitterionic SBMA in the A10-S15 and A15-S15 formulations significantly improved these values to 16.9 kPa of tensile strength with 90% of breaking elongation and 20.9 kPa of tensile strength with 120% of breaking elongation, respectively. Most notably, the further incorporation of hydrophobic LMA in A10-L5-S15 and A15-L5-S15 hydrogels produced remarkable mechanical reinforcement, achieving tensile strengths of 151 kPa and 158 kPa coupled with exceptional elongation at break values of 141% and 176%, respectively. These progressive improvements originated from synergistic molecular interactions including the electrostatic repulsion of quaternary ammonium groups, hydrogen bonding, along with the hydrophobic organization of LMA alkyl chains. Consequently, the integrated operation of these electrostatic repulsion, hydrogen bonding, and hydrophobic effects ultimately produces the desirable mechanical performance observed in the A-L-S hydrogel system.

Compression testing of hydrogels with varying compositions (Figure 3h) yielded results fully consistent with tensile measurements, where A15-L5-S15 demonstrated optimal performance with a compressive stress of 0.37 MPa at 80% strain.

### 3.4. Swelling of Hydrogels

**Figure 4:**
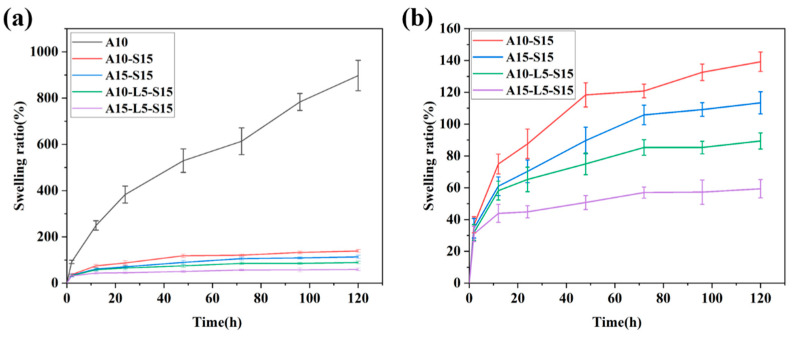
Swelling ratio curves of hydrogels for 120 h of swelling in DI water. (**a**) A10, A10-S15, A15-S15, A10-L5-S15, and A15-L5-S15 samples. (**b**) Magnification of A10-S15, A15-S15, A10-L5-S15, and A15-L5-S15 samples.

A quantitative analysis of swelling kinetics through weight measurements (Figure 4) revealed significant composition-dependent variations in hydrogel swelling behavior. The anti-swelling property refers to the ability of a hydrogel to resist excessive volume expansion in a solvent or to maintain its structural size stability after swelling. The A10 hydrogel displayed extensive swelling, reaching a swelling ratio of 800% after 120 h. The incorporation of zwitterionic SBMA consistently lowered the swelling ratios across all A-S hydrogels. This is because AA induced the protonation of SBMA, resulting in electrostatic repulsion force and thereby endowing them with anti-swelling properties. Most remarkably, the addition of hydrophobic LMA in both A10-L5-S15 and A15-L5-S15 hydrogels resulted in dramatically suppressed swelling, with the equilibrium swelling ratio decreasing to 89.4% and 59.36%, respectively, over the immersion period of 120 h.

In addition, we have also tested the anti-swelling properties of hydrogels at 4 °C and 50 °C (See Supplementary Materials Figure S1). The swelling rate of hydrogels was slower at 4 °C compared with that at 50 °C. After 2 h, the swelling ratio at 50 °C demonstrated a small decrease with the swelling time prolonged, reaching an equilibrium swelling ratio range of 33−70% after 120 h. In contrast, the hydrogels gradually swelled at 4 °C and demonstrated an equilibrium swelling range of 38−68% after 120 h. It can be found that the temperature can obviously change the swelling rate of hydrogels, but the equilibrium swelling ratio was not significantly affected. The anti-swelling properties under different temperatures indicate that our hydrogels possess good anti-swelling stability. This exceptional swelling resistance emerged from the synergistic combination of electrostatic repulsion and hydrophobic molecular interactions, creating effective barriers against water penetration. The good anti-swelling ability of hydrogels is advantageous for stable underwater sensing. Together, these electrostatic repulsion and hydrophobic effects produced an exceptionally dense A-L-S hydrogel network architecture capable of maintaining outstanding anti-swelling ability even during prolonged aqueous exposure.

### 3.5. Antibacterial Performance of Hydrogels

**Figure 5:**
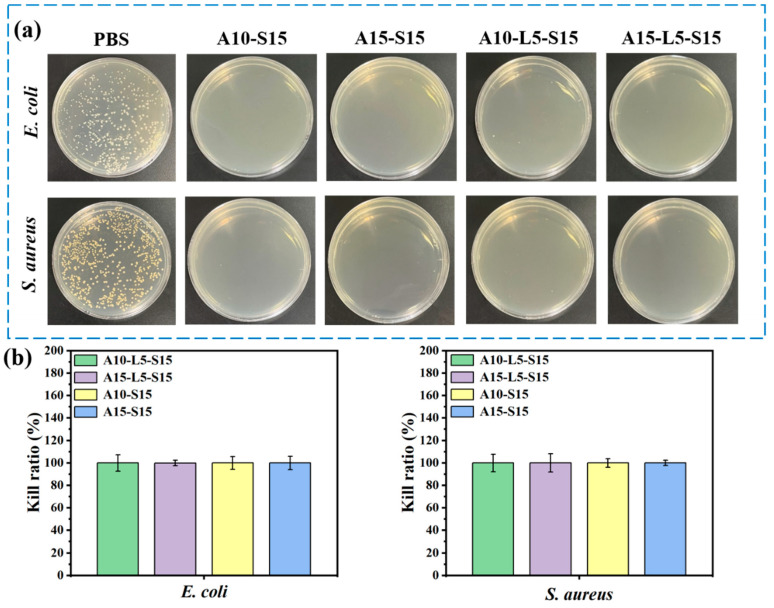
In vitro antibacterial activities of A-S and A-L-S hydrogels. (**a**) Schematic diagrams of antibacterial activities against *E. coli* or *S. aureus*. (**b**) Quantitative antibacterial efficiency against *E. coli* or *S. aureus*. PBS treated group was set as 0% of bacteria kill ratio.

Antibacterial functionality endows hydrogels with a protective barrier against bacteria threats. They address the significant biofouling challenge to long-term stable operation in biological environments. The antibacterial property enables hydrogel sensors to be used in harsh environments with extremely high hygiene requirements or where bacteria are easily grown. The hydrogels were evaluated against both Gram-negative (*E. coli*) and Gram-positive (*S. aureus*) bacteria following 1 h of co-culturing (Figure 5a). Following contact, bacterial suspensions were diluted and incubated for 24 h, revealing striking differences between the experimental and control groups. Bacterial colonies proliferated extensively throughout the control group, while hydrogel samples incorporating zwitterionic SBMA demonstrated exceptional antimicrobial performance, with virtually no viable bacterial colonies detected in the experimental groups. These pronounced antibacterial effects confirmed the successful integration of antimicrobial functionality into the hydrogel network, where the quaternary ammonium groups contributed to antibacterial activity.

The antibacterial performance of the hydrogels was quantitatively assessed through bacterial colony counting and calculation of the bacterial death ratio (Figure 5b). All A-S and A-L-S hydrogel formulations exhibited remarkable bactericidal efficacy, with SBMA-modified variants eliminating over 99% of both *E. coli* and *S. aureus*. This potent antibacterial activity was derived from multiple synergistic mechanisms. The cationic quaternary ammonium moieties in SBMA enable the electrostatic adsorption of anionic bacterial cells. subsequently disrupting their membrane integrity and inducing cell lysis. Furthermore, the weakly acidic microenvironment within the hydrogel network inhibited bacterial metabolic activity and growth [48], simultaneously promoting the hydrolysis of surface-exposed phospholipids and nucleic acids, collectively suppressing bacterial proliferation through these complementary pathways [49]. This excellent antibacterial activity has significant potential for applications in physiotherapy/rehabilitation.

### 3.6. Biocompatibility of Hydrogels

**Figure 6:**
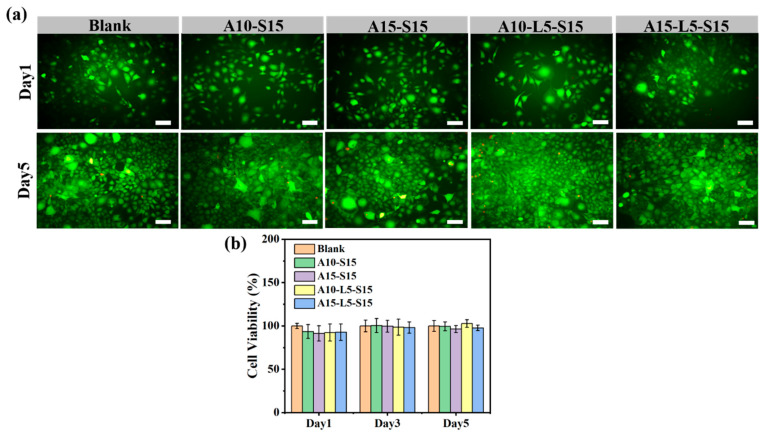
Biocompatibility evaluation of hydrogels co-cultured with L929 cells. (**a**) Live/dead fluorescence micrographs after 1 and 5 days of culture (scale bar = 200 μm). (**b**) Quantitative analysis of cell viability.

Biocompatibility refers to the capacity of a material to fulfill its designated purpose while avoiding detrimental immune reactions or toxic consequences in the host organism during biological interactions. Without biocompatibility, all ideas about wearable sensors are unrealistic. In this study, the cytocompatibility test was adapted to evaluate the biocompatibility of prepared A-S and A-L-S hydrogels. The cytocompatibility of the hydrogels was assessed using live/dead viability assays and CCK-8 analysis with L929 fibroblast cells. Before co-cultivation, A-S and A-L-S hydrogels were rinsed with culture medium to avoid toxicity. Microscopic observation after 1 and 5 days of co-culturing with A-S and A-L-S hydrogels revealed that cells maintained characteristic spindle-shaped morphology with distinct green fluorescence in live cell staining (Figure 6a). Throughout the culture period, cells exhibited well-defined contours without any apoptotic features such as membrane blebbing or cellular fragmentation. Notably, cell density increased significantly with prolonged culture duration. These collective findings demonstrated that neither the A-S nor A-L-S hydrogel formulations exerted cytotoxic effects on cells while simultaneously supporting normal cell proliferation and morphological integrity.

The CCK-8 assay provided a quantitative assessment of cellular viability, demonstrating that all hydrogel formulations maintained an excellent cell survival ratio exceeding 90% throughout the 1-, 3-, and 5-day incubation periods (Figure 6b). Collectively, these A-L-S hydrogels could be served as a highly biocompatible platform for biomedical applications.

### 3.7. Sensing Properties of Hydrogels

**Figure 7:**
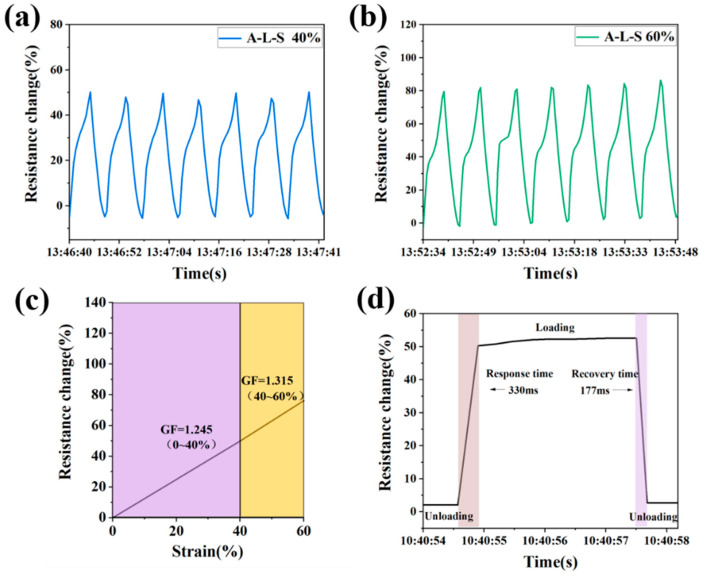
Relative resistance changes in A15-L5-S15 hydrogel with (**a**) 40% strain and (**b**) 60% strain. (**c**) Sensitivity of A15-L5-S15 hydrogel. (**d**) Response and recovery time of A15-L5-S15 hydrogel.

Building upon the exceptional antibacterial activity, mechanical property, and anti-swelling behavior of A15-L5-S15 hydrogels, we thoroughly characterized their strain-sensing capabilities under various deformation conditions (Figure 7a,b). The relative resistance change exhibited a progressive increase from approximately 50% to 80% as the applied tensile strain escalated from 40% to 60%, attributable to the gradual increase in conductive pathways during elongation. Notably, the resistance fully recovered to baseline values upon strain release, demonstrating excellent reversibility. The A15-L5-S15 hydrogel demonstrated an approximately linear response across varying strain ranges, achieving gauge factors (GFs) of 1.245 (0−40% strain) and 1.315 (40−60% strain) (Figure 7c), respectively. This ionic conductive hydrogel strain sensor demonstrated that the GF value increased with a higher strain range. The possible reason for this is that the main driver of resistance change shifts from macroscopic geometric deformation (small strain range) to a sharp decrease in ion transport efficiency (large strain range) caused by microstructural changes, leading to higher sensitivity in a large strain range. Moreover, the hydrogel system also attained remarkable response and recovery times, measured by 330 ms and 177 ms, respectively (Figure 7d). The performance of hydrogels with other anti-swelling hydrogels for sensing applications was compared (Table S1) [40,50,51,52,53,54,55,56]. Moreover, flexible materials can be combined with AI techniques to acquire, process, and analyze physiological signals, thereby providing feedback. Such systems not only improve signal quality but also enable devices to “understand” physiological states and respond intelligently, providing a model for the application of next-generation soft electronics platforms in personalized medicine and intelligent rehabilitation [57,58,59]. These comprehensive findings not only highlight the superior electromechanical performance of A15-L5-S15 hydrogels but also establish a platform for developing advanced flexible wearable electronics with combined high sensitivity and rapid response characteristics.

**Figure 8:**
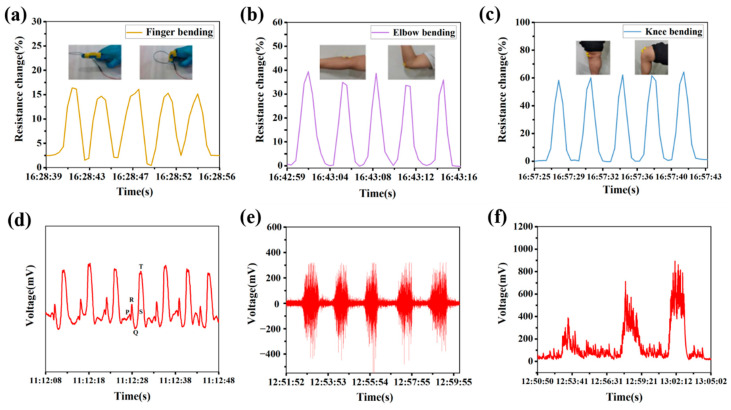
Human motion signal, EMG, and ECG monitoring of A15-L5-S15 hydrogel. (**a**) Finger bending. (**b**) Elbow bending. (**c**) Knee bending. (**d**) ECG signals. (**e**) EMG signals of muscle arm bending. (**f**) EMG signals of fist clenching under different intensities.

To comprehensively assess the sensing capabilities of A15-L5-S15 hydrogels, we directly attached them to various anatomical locations for human motion monitoring. When affixed to finger joints (Figure 8a), the hydrogel exhibited immediate resistance increases during finger flexion, responding precisely to motion deformation. For more substantial movements involving arms and knees, the A15-L5-S15 hydrogel consistently generated larger, reproducible resistance changes (Figure 8b,c). Recognizing the clinical significance of physiological signal monitoring, we further evaluated its capability in recording electrocardiogram (ECG) signal placements. As demonstrated in Figure 8d, the sensors captured all essential ECG components—including P-waves, QRS complexes, and T-waves—with exceptional waveform clarity and signal-to-noise ratios, confirming their efficacy for ECG signal monitoring. Similarly, during bicep contraction (Figure 8e), the hydrogel detected characteristic electromyogram (EMG) signal fluctuations that correlated with muscle tension, returning to baseline during muscle relaxation. A quantitative analysis of fist clenching at varying intensities revealed that the proportional signal amplitude increased with applied force (Figure 8f). Moreover, it can be assumed that combining hydrogel sensors with AI technology to build a closed-loop system from acquisition to analysis is the future direction for achieving real-time, accurate, and intelligent physiological monitoring [50,51,52]. Such systems not only improve signal quality but also enable devices to “understand” physiological states and respond intelligently, providing a model for the application of next-generation soft electronics platforms in personalized medicine and intelligent rehabilitation [14]. These collective results demonstrate that A15-L5-S15 hydrogels serve as high-performance sensing platforms, suitable for precisely tracking both human motion and physiological signals with rapid response and high stability.

## 4. Conclusions

In this study, anti-swelling ion-conductive hydrogels were constructed via free radical polymerization employing AA, LMA, and SBMA as monomers. The synergistic interactions of hydrophobic interactions and electrostatic repulsion forces within the hydrogel network yielded tunable anti-swelling properties, achieving an optimal equilibrium swelling ratio of just 59.36% after 120 h of aqueous immersion. These hydrogels demonstrated exceptional antibacterial performance and cytocompatibility, establishing their strong potential for flexible sensor applications. As a strain-sensing platform, the hydrogel exhibited gauge factors of 1.245 (0−40% strain) and 1.315 (40−60% strain), coupled with rapid response (330 ms) and recovery (177 ms) characteristics. Furthermore, the resultant hydrogel was capable of detecting and differentiating human physiological signals. In the future, the hydrogel can be integrated with AI-driven signal processing, improving signal resolution and identification in dynamic scenarios. This study is beneficial for developing a novel hydrogel platform for advanced health monitoring.

## Figures and Tables

**Table 1:** Primary composition of hydrogels.

Designation of Hydrogel	AA (μL)	LMA (μL)	SBMA (mg)	H_2_O (μL)
A10	100	/	/	1000
A10-S15	100	/	150	1000
A15-S15	150	/	150	1000
A10-L5-S15	100	50	150	1000
A15-L5-S15	150	50	150	1000

## Data Availability

The data presented in this study are available on request from the corresponding author.

## Supplementary Materials

The following supporting information can be downloaded at: https://www.mdpi.com/article/10.3390/jfb16090346/s1, Figure S1. Swelling ratio curves of hydrogels for 120 h of swelling in DI water at (a) 4 °C and (b) 50 °C. Table S1. Results comparison of related anti-swelling hydrogels.
