# Supplementary material for: Anti-Swelling Antibacterial Hydrogels Based on Electrostatic Repulsion and Hydrophobic Interactions for Human Motion Sensing
Source: J Funct Biomater. 2025 Sep 14;16(9):346. doi: 10.3390/jfb16090346 (PMC12470998; doi:10.3390/jfb16090346)
Supplement: Supplementary file 1 [file jfb-16-00346-s001.zip › jfb-3783003-supplementary.pdf]

# Anti-swelling antibacterial hydrogels based on electrostatic repulsion and hydrophobic interactions for human motion sensing

Zexing Deng [a, 1], Litong Shen [a, 1], Qiwei Cheng [a], Ying Li [a], Tianming Du [b, *], Xin Zhao [c, *]

[a] *College of Materials Science and Engineering, Xi'an University of Science and Technology, Xi'an, 710054, China*

[b] *Beijing International Science and Technology Cooperation Base for Intelligent Physiological Measurement and Clinical Transformation, Department of Biomedical Engineering, College of Chemistry and Life Science, Beijing University of Technology, Beijing 100124, China.*

[c] *State Key Laboratory for Mechanical Behavior of Materials, Xi'an Jiaotong University, Xi'an, 710049, China*

[1] These authors contributed equally to this work.

Correspondence: Tianming Du, email: dutianming@bjut.edu.cn; Xin Zhao, email: zhaoxinbio@mail.xjtu.edu.cn

Table S1. Results comparison of related anti-swelling hydrogel.

| Materials | Anti-swelling property | Antibacterial | GF | Ref. |
|---|---|---|---|---|
| PVA-Gp/TA-$CaCl_2$ | 89% of swelling ratio after 30 days | √ | 1.73 | 1 |
| PVA/AMY | 28.6% of swelling ratio after 20 days | – | 2.1-4.96 | 2 |
| PVA/poly(SBMA-HEMA) | 9% of swelling ratio after 30 days | – | 1.43-3.36 | 3 |
| *t*-BuA/DMAA/IL/[BMIm]TFSI | 3.8% of swelling ratio after 10 days | – | 1.06-1.30 | 4 |
| AA/LMA/CTAB | ~4% of swelling ratio after 15 days | – | 0.42-7.39 | 5 |
| AA-MEA-Fe/CS | ~16% of swelling ratio after 7 days | – | 1.60-2.57 | 6 |
| PAA-CS-$Al^{3+}$-MXene | 3.8% of swelling ratio after 7 days | – | 4.7-63.0 | 7 |
| PVA/P(AM-VBIPS) | 1% of swelling ratio after 1 day | – | 1.99-2.88 | 8 |
| Poly(AA-LMA-SBMA) | 59.4% of swelling ratio | √ | 1.24 | Thi |

| | | |
|---|---|---|
| after 5 days | -1.32 | s work |

PVA: Polyvinyl alcohol; Gp: β-Glycerophosphate sodium; TA: Tannic acid; AMY: Amylopectin; HEMA: 2-hydroxyethyl methacrylate; *t*-BuA: *tert*-butyl acrylate; DMAA: N,N-dimethylacrylamide; IL: 1-vinyl-3-butylimidazolium; [BMIm]TFSI: bis(trifluoromethanesulfonyl) imide; CTAB: Cetyltrimethylammonium bromide; MEA: Ethylene glycol methyl ether acrylate; CS: Chitosan; PAA: Poly(acrylic acid); PAM: Polyacrylamide; VBIPS; 3-(1-(4-vinylbenzyl)-1H-benzo-[d]imidazole-3-ium-3-yl) propane-1-sulfonate; "–" means not determined or detected.

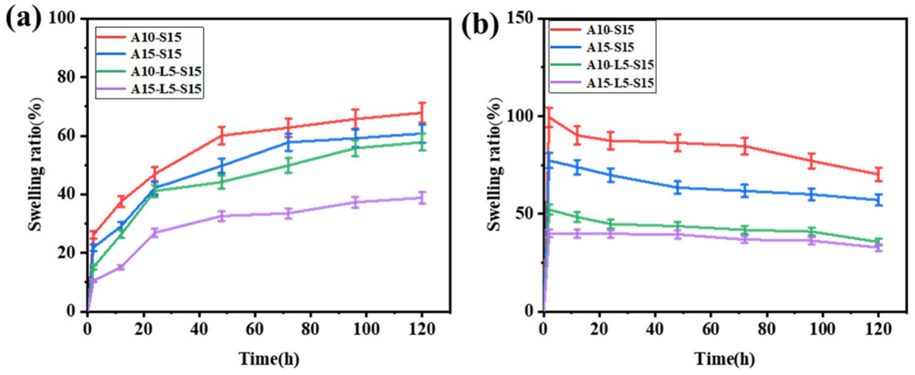


**Figure S1.** Swelling ratio curves of hydrogels for 120 hours of swelling in DI water at (a) 4 °C and (b) 50 °C.

## References


[1] Chen, K.; Liang, K.; Liu, H.; Liu, R.; Liu, Y.; Zeng, S.; Tian, Y. Skin-inspired ultra-tough supramolecular multifunctional hydrogel electronic skin for human-machine interaction. *Nano-Micro Lett.* **2023**, *15*, 102.

[2] Gao, Y.; Wang, Y.; Dai, Y.; Wang, Q.; Xiang, P.; Li, Y.; Gao, G. Amylopectin based hydrogel strain sensor with good biocompatibility, high toughness and stable anti-swelling in multiple liquid media. *Eur. Polym. J.* **2022**, *164*, 110981.

[3] Ren, J.; Liu, Y.; Wang, Z.; Chen, S.; Ma, Y.; Wei, H.; Lü, S. An anti-swellable hydrogel strain sensor for underwater motion detection. *Adv. Funct. Mater.* **2021**, *32*, 2107404.

[4] Wei, J.; Zheng, Y.; Chen, T. A fully hydrophobic ionogel enables highly efficient wearable underwater sensors and communicators. *Mater. Horiz.* **2021**, *8*, 2761-2770.

[5] Qi, C.; Dong, Z.; Huang, Y.; Xu, J.; Lei, C. Tough, anti-swelling supramolecular

hydrogels mediated by surfactant-polymer interactions for underwater sensors. *ACS Appl. Mater. Interfaces* **2022**, *14*, 30385-30397.

[6] Zhao, Z.; Qin, X.; Cao, L.; Li, J.; Wei, Y. Chitosan-enhanced nonswelling hydrogel with stable mechanical properties for long-lasting underwater sensing. *Int. J. Biol. Macromol.* **2022**, *212*, 123-133.

[7] Huang, H.; Shen, J.; Wan, S.; Han, L.; Dou, G.; Sun, L. Wet-adhesive multifunctional hydrogel with anti-swelling and a skin-seamless interface for underwater electrophysiological monitoring and communication. *ACS Appl. Mater. Interfaces* **2023**, *15*, 11549-11562.

[8] Wang, S.; Zhang, D.; He, X.; Zhou, J.; Zhou, Y.; Wang, X.; Wang, Z.; Liu, S.; Zheng, S.Y.; Yang, J. Anti-swelling zwitterionic hydrogels as multi-modal underwater sensors and all-in-one supercapacitors. *ACS Appl. Polym. Mater.* **2022**, *4*, 7498-7507.
